# TET1 regulates fibroblast growth factor 8 transcription in gonadotropin releasing hormone neurons

**DOI:** 10.1371/journal.pone.0220530

**Published:** 2019-07-30

**Authors:** Megan L. Linscott, Wilson C. J. Chung

**Affiliations:** 1 Department of Biological Sciences, Kent State University, Kent, Ohio, United States of America; 2 School of Biomedical Sciences, Kent State University, Kent, Ohio, United States of America; Barts and The London School of Medicine and Dentistry Blizard Institute, UNITED KINGDOM

## Abstract

Fibroblast growth factor 8 (FGF8) is a potent morphogen that regulates the ontogenesis of gonadotropin-releasing hormone (GnRH) neurons, which control the hypothalamus-pituitary-gonadal (HPG) axis, and therefore reproductive success. Indeed, FGF8 and FGFR1 deficiency severely compromises vertebrate reproduction in mice and humans and is associated with Kallmann Syndrome (KS), a congenital disease characterized by hypogonadotropic hypogonadism associated with anosmia. Our laboratory demonstrated that FGF8 signaling through FGFR1, both of which are KS-related genes, is necessary for proper GnRH neuron development in mice and humans. Here, we investigated the possibility that non-genetic factors, such as the epigenome, may contribute to KS onset. For this purpose, we developed an embryonic explant model, utilizing the mouse olfactory placode (OP), the birthplace of GnRH neurons. We show that TET1, which converts 5-methylcytosine residues (5mC) to 5-hydroxymethylated cytosines (5hmC), controls transcription of *Fgf8* during GnRH neuron ontogenesis. Through MeDIP and ChIP RT-qPCR we found that TET1 bound to specific CpG islands on the *Fgf8* promoter. We found that the temporal expression of *Fgf8* correlates with not only TET1 binding, but also with 5hmC enrichment. siRNA knockdown of *Tet1* reduced *Fgf8* and *Fgfr1* mRNA expression. During this time period, *Fgf8* also switched histone status, most likely via recruitment of EZH2, a major component of the polycomb repressor complex-2 (PRC2) at E13.5. Together, these studies underscore the significance of epigenetics and chromatin modifications to temporally regulated genes involved in KS.

## Introduction

Fibroblast growth factors (FGFs) are well-known signaling proteins that are crucial for neuronal fate specification, progenitor cell proliferation, and cell survival [1–7]. In the developing brain, FGF8 is required for proper formation of the midbrain-hindbrain, telencephalon, midline structures, cerebellum, and the olfactory placode (OP) [8–12]. Indeed, inactivation of FGF8 function results in malformation of various brain regions [13–18]. As such, location and dosage of *Fgf8* mRNA expression is critical for initiating developmental cellular responses, as they elicit downstream signaling factors, which in turn, establish patterning and orientation of brain regions [11,17,19–22].

Our previous studies showed that FGF8 signaling is required for gonadotropin-releasing hormone (GnRH) neuron ontogenesis in the OP [2,3,5,14]. However, it is still not understood what drives *Fgf8* transcription in the developing OP. Several studies showed that multiple downstream, conserved DNA sequences could recapitulate *Fgf8* expression patterns in both zebrafish and mouse embryos [23–25]. These studies suggested chromosomal conformation is dynamically regulated in the context of tissue type, and that both activating and repressive *cis*-regulatory elements can drive *Fgf8* expression. However, few studies have addressed the chromatin state of the *Fgf8* locus, which is likely to play a role in cis-regulatory enhancer-promoter interactions, or alternatively, how the epigenomic state may control the temporal regulation of *Fgf8* transcription.

**Fig 1:**
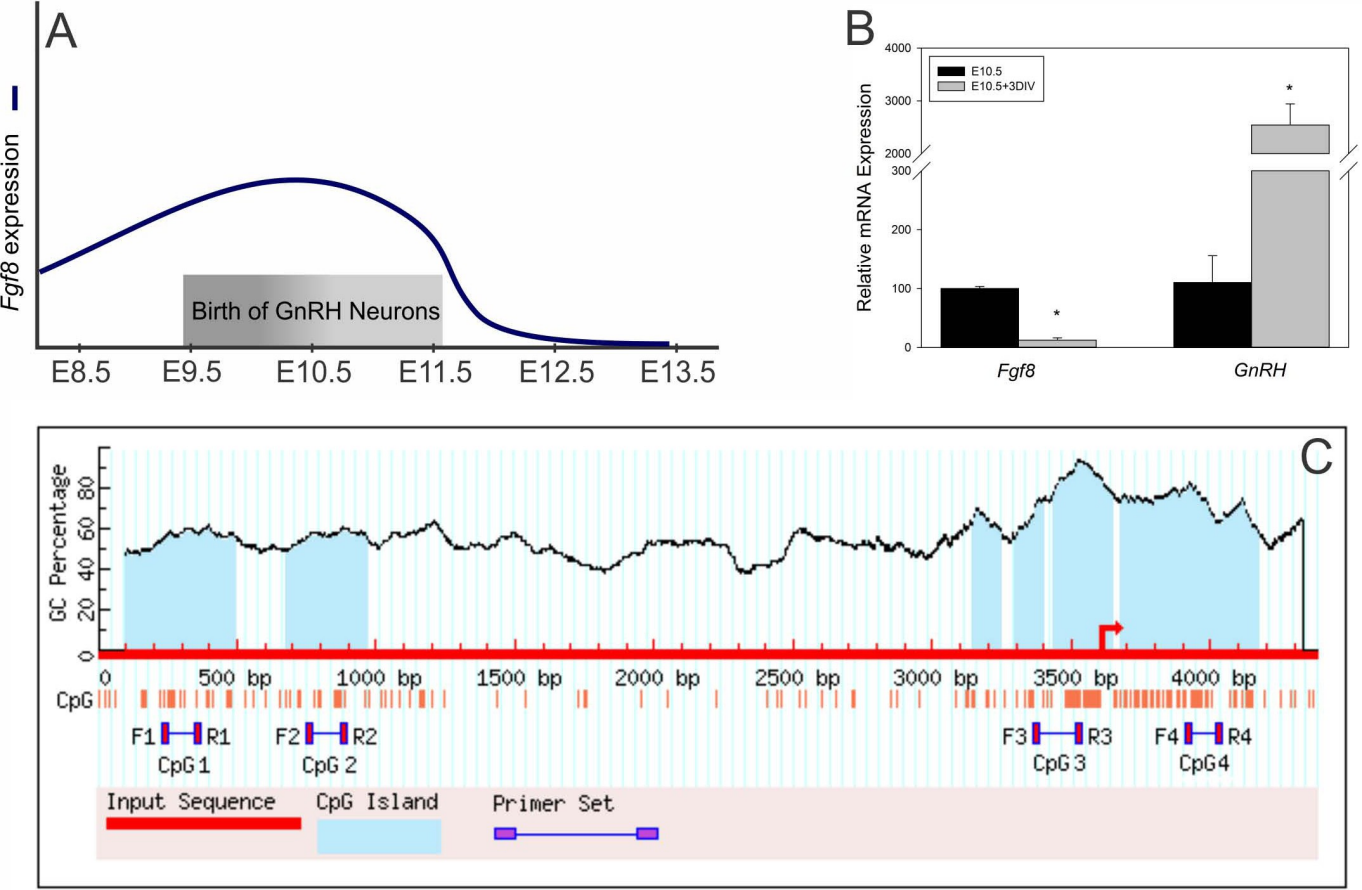
**A) Schematic of transient *Fgf8* transcription during GnRH neuronal emergence in the embryonic mouse OP**. B) *Fgf8* and *GnRH* mRNA expression at E10.5 (n = 2) or at E10.5+3 days *in-vitro* (DIV) (n = 3) in the mouse OP. *p* < 0.0005, *p* < 0.01; Student’s *t*-test C) MethPrime CpG prediction of CpG islands on mouse *Fgf8* promoter with relative locations of CpG1-4 primers [30]. * indicates *p* <0.05.

Our recently published results studied *Fgf8* gene transcription during emergence of GnRH neurons in the embryonic day (E) E9.5-E13.5 OP, a heterogeneous cell population of neurons and epithelial cells, that contributes to GnRH neuron proliferation and differentiation [26–28] (Fig 1). In these studies, we found that *Fgf8* transcription may be under the direct control of DNA methylation [29]. Interestingly, we found that the *Fgf8* promoter region and gene body harbors three major CpG islands upstream (CpG 1, 2, 3) and one downstream of the translation start site (TSS; CpG 4) of the *Fgf8* gene. Moreover, our experimental data showed that the DNA methyltransferase (DNMT) inhibitor, 5-azacitidine (AZA), induced *Fgf8* mRNA expression in GT1-7 GnRH neurons [28]. Collectively, the results from these initial studies led to the premise that the upregulation of *Fgf8* transcription in the embryonic mouse OP may be a DNA methylation-dependent process.

To test our hypothesis, we focused on two mechanisms that contribute to methylation: CpG dinucleotide methylation and histone modifications. The ability of DNA and histone methylating proteins to modify chromatin architecture is a key developmental regulatory component, which maintain genes in an active or inactive state, and control cell fate decisions. In general, DNA methylation changes are catalyzed by DNMTs, which convert cytosines to 5-methylcytosine (5mC), and methylated DNA can be demethylated by ten-eleven translocation methylcytosine dioxygenases (TETs), which convert 5mC to 5-hydroxymethylcytosine (5hmC) [31]. In neurons, the transition from progenitor to a differentiated neuron is associated with high levels of 5hmC [32–34]. At the genomic level, it is well known that 5hmC associates with genes important for neuronal function and correspond with gene transcription [32,35,36]. However, it is unclear how neural OP progenitor cells coordinate the process of DNA demethylation of the *Fgf8* locus during development and what factors are required for this process.

As previously indicated, the methylation pattern of histones is of equal importance in respect to *Fgf8* transcription. Specifically, two modifications, H3K27me3, a repressive histone mark, and H3K4me3, an activating histone mark, form bivalent domains which poise neurogenic genes for activation, and are thought to respond to developmental cues. Chromatin-associated proteins, such as the polycomb repressive complex 2 (PRC2), which contribute to H3K27 trimethylation, are also important for developmental gene regulation, as they have been shown to regulate proliferation, neurogenesis, WNT signaling, cell cycle exit, and compaction of chromatin [37]. Interestingly, mice mutant for *Fgf8* or PRC2 genes (i.e., histone-lysine N-methyltransferase *Ezh2*) share a common phenotype in which embryos fail to undergo gastrulation and have reported proliferation defects [38–40]. Additionally, *Fgf8* is suppressed in the trunk tissue of developing embryos [41] by a mechanism that recruits PRC2 proteins. These studies suggest a mechanism for *Fgf8* transcription via local histone modifications that may affect the accessibility of the chromatin. However, little is known about whether PRC2 proteins also regulate OP *Fgf8* transcription during GnRH neuron emergence, and whether they contribute to GnRH neurobiology.

Here we show that *Fgf8* expression in the developing mouse OP is under the control of epigenetic switches involving both DNA and histone-modifying proteins. Specifically, we studied epigenetic control of *Fgf8* transcription in the E9.5—E13.5 mouse OP, which is known to have high *Fgf8* transcriptional activity. First, we examined DNMT and TET mRNA expression as well as protein-DNA binding profiles, and whether inhibition of DNMTs upregulated *Fgf8* mRNA in embryonic OP explants. Second, we investigated 5hmC conversion via TET1 on CpG islands along the *Fgf8* promoter during OP cell differentiation. Specifically, we focused on TET1, since unlike the other TETs, it is associated with gene promoters [42]. Third, we demonstrated that *Fgf8* histone modifications, along with other DNA modifiers, are essential for the precise timing of *Fgf8* mRNA production during mid-gestational mouse development. Together, we show that the state of *Fgf8* chromatin contributes to temporally-dependent regulation of transcriptional activity.

## Materials and methods

### Timed-breeding of mice and nasal explant cultures

Adult wildtype 129P2/OlaHsd*CD-1 male x female mice were timed-bred in the late afternoon in our animal facility (12L:12D cycle) with access to food and water *ad libitum*. All procedures were approved by the Institutional Animal Care and Use Committee at Kent State University. In the morning, females with a sperm plug were denoted as embryonic day (E) 0.5. Adult pregnant female 129P2/OlaHsd*CD-1 mice were sacrificed at E9.5, E10.5 or E13.5. The uterine horns were quickly removed from the mice, and kept in sterile ice-cold phosphate-buffered saline (Sigma-Aldrich, P3813). Following, the nasal region containing the OPs was surgically isolated using a dissection microscope, placed on 0.65 μm Durapore membrane filters (Millipore, DVPP04700), transferred to cell tissue culture inserts (Corning, 353095), and grown using the liquid-air interphase method with in phenol-red free Dulbecco's modified Eagle's medium (DMEM)/F12/glutamax (Thermofisher Scientific, 10565018) supplemented with B27 (Thermofisher Scientific, 17504044) and 1% pen/strep/myc (Sigma-Aldrich, A5955) media. For expression assays of TETs and DNMTs, Chromatin immunoprecipitation, and MeDIP experiments, E9.5, E10.5, or E13.5 nasal explant tissues were collected, immediately flash frozen and kept at -80°C.

### Cell culture

Immortalized mouse GnRH GT1-7 and GN11 neurons (generously donated by Dr. Pamela Mellon, University of San Diego, CA; generously donated by Dr. Sally Radovick, Robert Wood Johnson Medical School, NJ) were grown in phenol-red free DMEM containing 4.5 g/L pyruvate and 548 mg/L-glutamine, 10% fetal bovine serum (ATCC, 30–2020), 1% pen/strep (Sigma-Aldrich, A5955) [43]. Cells were kept in a humidified incubator at 37°C with 5% CO_2_.

### Pharmacological treatments

OP explants were treated for 72 h (i.e., 3 days *in vitro* (DIV)) in the presence of vehicle (0.005% DMSO) or 1 μM 5-azacitidine (AZA) (Tocris Biosciences, 3842). Our AZA dose and length of treatment was based on previous studies showing that these conditions were able to induce gene expression [44,45]. Furthermore, AZA was able to induce *Fgf8* mRNA expression in GT1-7 neurons from our previous studies [28]. Total cellular RNA extraction and cDNA synthesis were performed as described below.

### RT-qPCR

Total cellular RNA was extracted with TriPure (Roche, 11667165001) according to the manufactures instructions. RNA purity and concentration was measured using the Synergy H2 multi-mode reader with a Take3 Micro-Volume plate adapter (Biotek). ProtoScript II First Strand cDNA Synthesis Kit (New England Biolabs, E6560L). ProtoScript II First Strand cDNA Synthesis Kit was used to reverse transcribe 0.5 μg of total RNA. RT-qPCR was performed in triplicate with gene-specific, intron-spanning primers (Table 1) using a Mastercycler EP Realplex^2^ (Eppendorf, EPPE6300000.604) with SYBR Green PCR Master Mix (Roche, 04707516001). Relative mRNA expression levels were calculated using the ΔΔ^-2CT^ method [46]. Hypoxanthine phosphoribosyltransferase 1 (*Hprt-1*) was used as a housekeeping gene.

**Table 1:** Forward and reverse primer sequences for detection of genomic DNA and mRNA transcripts used in this study.

Target	Forward (5'-3')	Reverse (5'-3')	bp
DNA			
Fgf8 CpG 1	AACTGCTCGTGGTCGTACAG	GTGCCCCCAACTAACTCCTC	152
Fgf8 CpG 2	GGTGGACGTCGAGCACAG	AAGGGCTATCCCGAAAAGGTG	156
Fgf8 CpG 3	ACATTAGGCGACCCAGAGAC	CGGGATCGTCCAGGGATTG	144
Fgf8 CpG 4	GGTACAAGGGCAATGGGGAC	CACCTTACCGAAGGGGTCTC	275
Fgf8-non-specific	GTCAGTCTGCGAATATAGCTCAG	CACAGTACCAACAAGTGTCACAG	314
Fgf8 3'UTR	CCCAACTACCTGCAGAGCAA	TTGAGGAACTCGAAGCGCAG	242
mRNA			
Tet1	ACAAAAAGCGTACCTGCACC	CCGGTTTTCACGTCACTTCC	214
Tet2	AGGGACCAGAACCAGGCT	TTGAATGAATCCAGCAGCACC	171
Tet3	CCTCGGCGGGGATAATGG	ACGAGCATTTATTTCCACCTCG	78
DNMT3a	GAGCCGCCTGAAGCCC	TTTCGATCATCCTCCCGCTC	230
DNMT3b	ATCCATAGTGCCTTGGGACC	CTCCTGTCATGTCCTGCGT	294
DNMT1	GTACATGCTGCTTCCGCTTG	CAAGTCTTTGAGCCGCCTG	197
Fgf8	AGAAGACGGAGACCCCTTCG	TGAATACGCAGTCCTTGCCTT	158
GnRH	GGCATTCTACTGCTGACTGTGT	CTACATCTTCTTCTGCCTGGCT	252
Fgfr1	ATGGTTGACCGTTCTGGAAG	TGGCTATGGAAGTCGCTCTT	171
Hprt	CTCATGGACTGATTATGGACAGGAC	GCAGGTCAGCAAAGAACTTATAGCC	123

### Methylated immnuoprecipation (MeDIP)

Olfactory placodes from 4–6 E9.5, E10.5, and E13.5 embryos were treated with RNaseA, lysed overnight and isolated with phenol chloroform. 5 μg of DNA was sonicated to 500–200 bp. DNA was incubated at 95°C for 10 minutes and quickly placed on ice. Dynabeads (Invitrogen, 11203D) were incubated for 2 hours with salmon sperm and washed with PBS-T. DNA (800 ng) was immunoprecipitated with 1.25 μg of either rabbit anti-5mC (Active Motif, 61255), rabbit anti-5hmC (Active Motif, 39791), or normal rabbit IgG (Millipore, 12–370), and 8 ng (1%) of the precipitated DNA was saved as input control. Following incubation, DNA was incubated for 4 hours at 4°C on a 3D rotator with the respective antibodies. Beads were pelleted using a magnet and washed in PBS and TE buffer on a 3D rotator for 10 minutes at 4°C. Antibodies were digested with PK at 60°C for 2 hours followed by a 10 minute 95°C PK inactivation step. Samples were subsequently purified using phenol-chloroform and glycogen. Each sample was ran on an Eppendorf RT-qPCR program with primers flanking 4 CpG islands on the *Fgf8* gene. Relative enrichment was calculated using the percent input method, where each immunoprecipitation is adjusted to input loading controls, and compared to negative control IgG for significant 5mC or 5hmC enrichment. Non-specific primers flanking upstream of the of *Fgf8* were used as negative control in the DNMT3b ChIP.

### Chromatin immunoprecipitation (ChIP)

ChIP was used to examine histone modifications (H3K4me3 and H3K27me3) along with TET1, DNMT3a, DNMT3b and DNMT1, and EZH2 interaction along the promoter region of *Fgf8*. Embryos were dissected at E9.5, E10.5 or E13.5, and pooled in microcentrifuge tubes containing 4–6 OPs per sample. Chromatin was harvested using the EZ-Magna-ChIP kit (Millipore, 17–10086) according to manufacturer's instructions. Briefly, cells were cross-linked with 1% formalin for 10 min and lysed. The protein cross-linked genomic DNA was fragmented to 200–600 base pairs using sonication. All chromatin samples were verified for correct shearing density on an agarose gel before continuing. Magnetic Protein A/G beads were blocked with salmon sperm for 2 hours, and washed in PBS. Following, the fragments were immunoprecipitated using 1.25 μg of either rabbit polyclonal antibody against DNMT3b, DNMT1, DNMT3a (Abcam, ab2851, ab13537, ab2850), EZH2, TET1, H3K4me3, and H3K27me3 (Active Motif, 39901, 61443, 39915, 39155) or control IgG (Millipore, 12–370) for 3 hours at 4°C on a 3D rotator. Magnetic beads were pulled out of solution using a magnet, and bound fragments were washed four times for ten minutes. Proteinase K (10 mg/ml) was used to reverse crosslinking, and DNA was isolated using phenol chloroform. The relative amount of protein occupancy on the identified sites on the promoter of *Fgf8* was measured using a Mastercycler EP Realplex^2^ (Eppendorf, EPPE6300000.604) with SYBR Green PCR Master Mix (Roche, 04707516001). For this purpose, four primer sets were designed to flank the CpG islands on the promoter region and an intragenic site of *Fgf8*. ChIP signal was measured using the percent input method as described above. Non-specific primers flanking the 3’UTR of *Fgf8*, a CpG poor region, were also used in the MeDIP (S1 Fig).

### Tet1 siRNA treatment

siRNA experiments were conducted using Accell SMARTpool siRNA, which targets 4 portions of the *Tet1* mRNA transcript (CUAUUUGUCUAUUAUGUGUG, UCGUUGGGUCUAAAGGCUU, UCGCUAAACUAACUAUAAAUGUAU, UUAUAGUUUUAAAUACUUA). A *non-targeting* siRNA pool was used as a negative control (UGGUUUACAUGUCGACUAA, UGGUUUACAUGUUUUCUGA,UGGUUUACAUGUUUUCCUA, UGGUUUACAUGUUGUGUGA). All siRNAs were resuspended in 5x siRNA buffer diluted in RNase free water. GT1-7 hypothalamic neurons were seeded in 24-well plates in DMEM and FBS with 1% penicillin/streptomycin. Growth medium was removed and replaced with Accell delivery media (Dharmacon, B-005000-100) with 1 μM *Tet1* siRNA (Dharmacon, E-06861-00-0020) or *non-targeting* control (Dharmacon, D-001910-20-20). After 3 days, cells were collected for RNA as described previously.

### 5mC and 5hmC dot blot

Dot blot experiments were used to determine genome-wide changes in DNA. Total DNA was isolated as previously described. 150ng and 75ng of DNA were diluted in 20x SSC, heated at 95°C, cooled on ice, and blotted on a nitrocellulose membrane soaked in 10x SSC. The membrane was UV crosslinked for 10 minutes on a Benchtop UV Transilluminator (UVP, M-20V). Following, the membrane was blocked in 5% dry milk and 2% normal sheep serum for 1 hour, before an overnight incubation (1:5000) with 5hmC or 5mC (Active Motif, 39791, 61255) in blocking solution. The membrane was washed three times in TBS-Tween and incubated in peroxidase anti-rabbit IgG (1:2000)(Vector Laboratories, PI-1000) for one hour, and imaged using Clarity Western ECL substrate (BioRad, 1705061). Images were processed and quantified using ImageJ studio.

### Statistical analysis

Data were analyzed using Student *t*-tests or one-way analysis of variance (ANOVA) with treatment and/or DIV between subject variables. Holm-Sidak method tests were used for *post-hoc* analysis. Group numbers (n) are given in the figure legends. Differences were considered significant if *p* < 0.05.

## Results

### Identification of CpG islands on the *Fgf8* promoter

To identify CpG islands along the length of the *Fgf8* promoter, we analyzed 5000 basepairs along the 5’UTR and an intragenic site localized between exons 1 and 2 using MethPrimer [30]. Here, we found 4 major CpG islands, with 3 located in the 5’ UTR and 1 located intragenically (Fig 1C).

### Inhibition of DNMT activity increases *Fgf8* expression in the embryonic mouse OP

**Fig 2:**
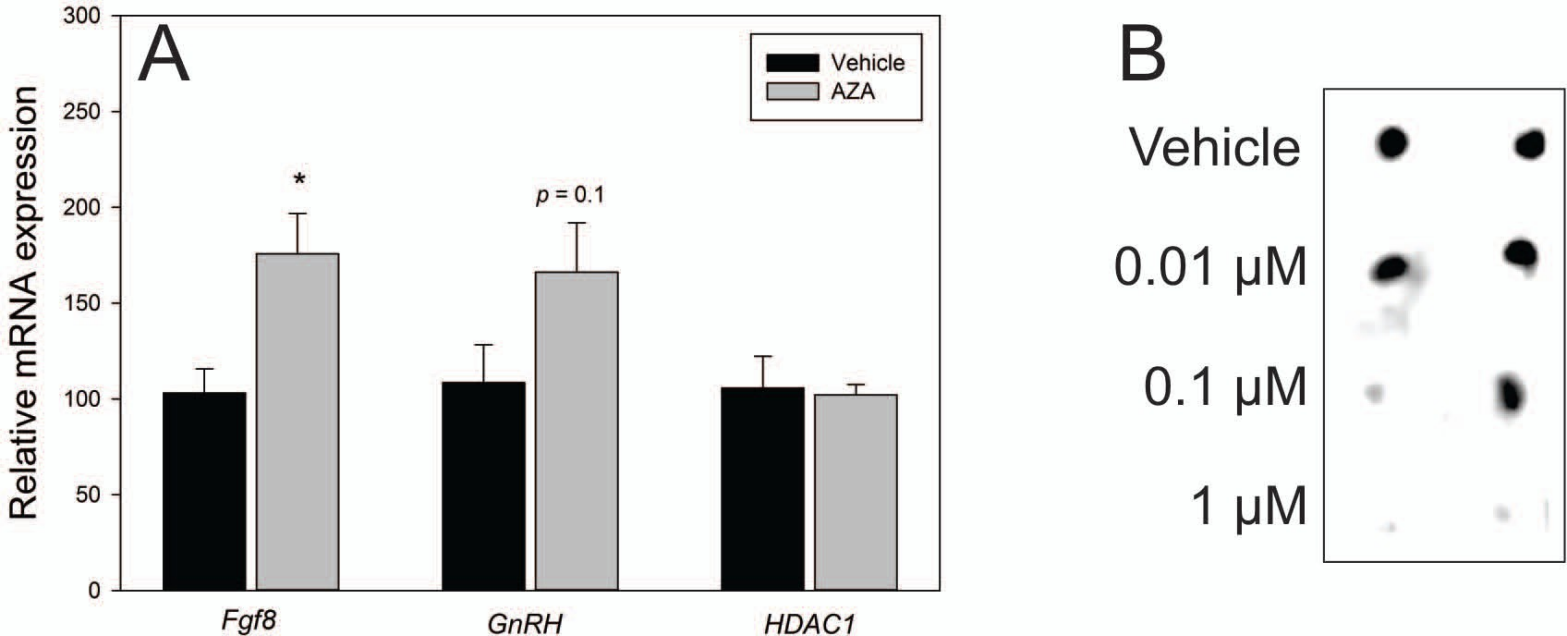
AZA induced *Fgf8* expression in the OP. A) RT-qPCR for *Fgf8*, *GnRH* and *HDAC1* mRNA in vehicle (n = 6) vs 1 μM AZA-treated E10.5 mouse OP explants (n = 6) for 3 DIV B) Two representative 5mC dot blots in GT1-7 neurons treated with AZA (Vehicle, 0.01, 0.1, or 1 μM) for 3 days. Note that 5mC is virtually absent at the 1 μM concentration only. * indicates *p* < 0.05; Student’s *t*-test.

Our previous studies in GT1-7 neurons revealed that inhibition of DNMT activity using AZA, upregulated *Fgf8* expression. Here, we found that AZA treatment (1 μM) in E10.5 OP explants cultured 3 DIV increased *Fgf8* mRNA expression (*p* = 0.02; Fig 2A). Contrary to what was found previously in the GT1-7 cell line, the increase in OP *GnRH* mRNA expression was not significant (*p* = 0.1; Fig 2A) [28]. Dot blot analysis of overall 5mC levels in GT1-7 cells showed a decrease with increasing AZA concentration (Fig 2B). Propidium iodine staining in GT1-7 neurons showed no significant changes in cell death when treated with 1 μM AZA 3 DIV (*p* = 0.5), (S2 Fig).

### Tet and DNMT expression in the OP

**Fig 3:**
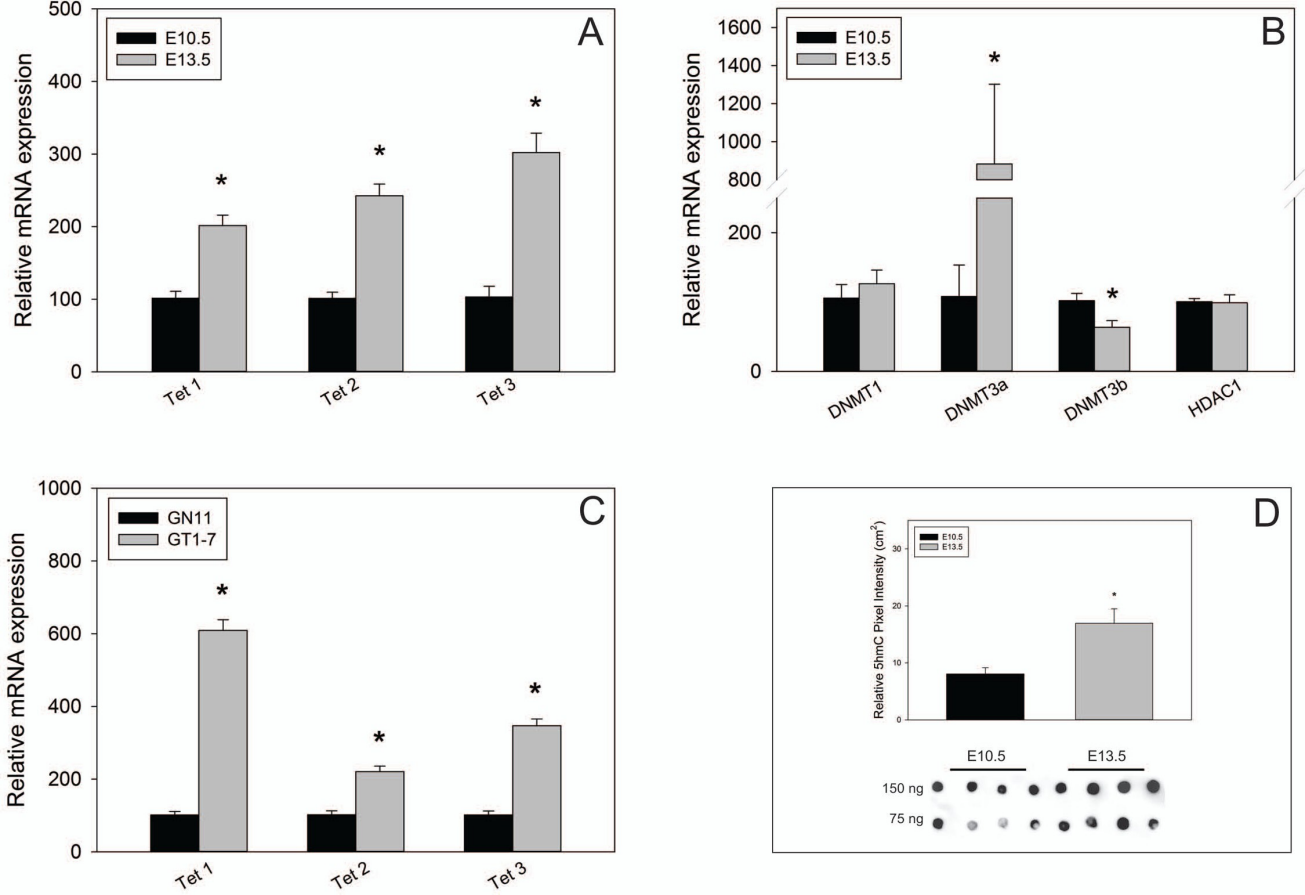
*TET or DNMT* expression in the OP. A) *Tet 1*, *2*, *3* or B) *Dnmt1*, *3a*, *3b*, *and HDAC1* mRNA expression in E10.5 or E13.5 mouse OP (n = 4). C) *Tet 1*, *2*, *3* mRNA expression in GN11 and GT1-7 GnRH neurons (n = 4). D) 5hmC dot blot quantification of E10.5 (n = 4) versus E13.5 (n = 4) in 75 ng of OP genomic DNA and original dot blot image. * indicates *p* < 0.05; Student’s *t*-test.

To determine the transcriptional activity of DNA methylation modifiers in the embryonic OP, we analyzed their mRNA levels in E10.5 and E13.5 OPs. We showed that *Tet1*, *Tet2*, and *Tet3* mRNA was increased in E13.5 OPs compared to E10.5 OPs (*p* = 0.001, *p* = 0.0002, *p* = 0.0006 respectively; Fig 3A). *Dnmt3a* mRNA increased 10-fold (*p* = 0.006), while *Dnmt3b* mRNA levels significantly decreased (*p* = 0.03; Fig 3B). No significant changes were detected for *Dnmt1* or *Hdac1* mRNA expression (*p =* 0.5, *p* = 0.9). We further found that *Tet1*, *Tet2*, and *Tet3* mRNA expression was also higher in developmentally mature GT1-7 compared to migratory, immature GN11 neurons (*p* = 0.000003, *p* = 0.0007, *p* = 0.00002; Fig 3C). Moreover, 5hmC DNA dot blot experiments showed that 5hmC levels are significantly higher in E13.5 compared to E10.5 OPs (*p* = 0.01; Fig 3D).

### DNMT3b interacts with the *Fgf8* locus

**Fig 4:**
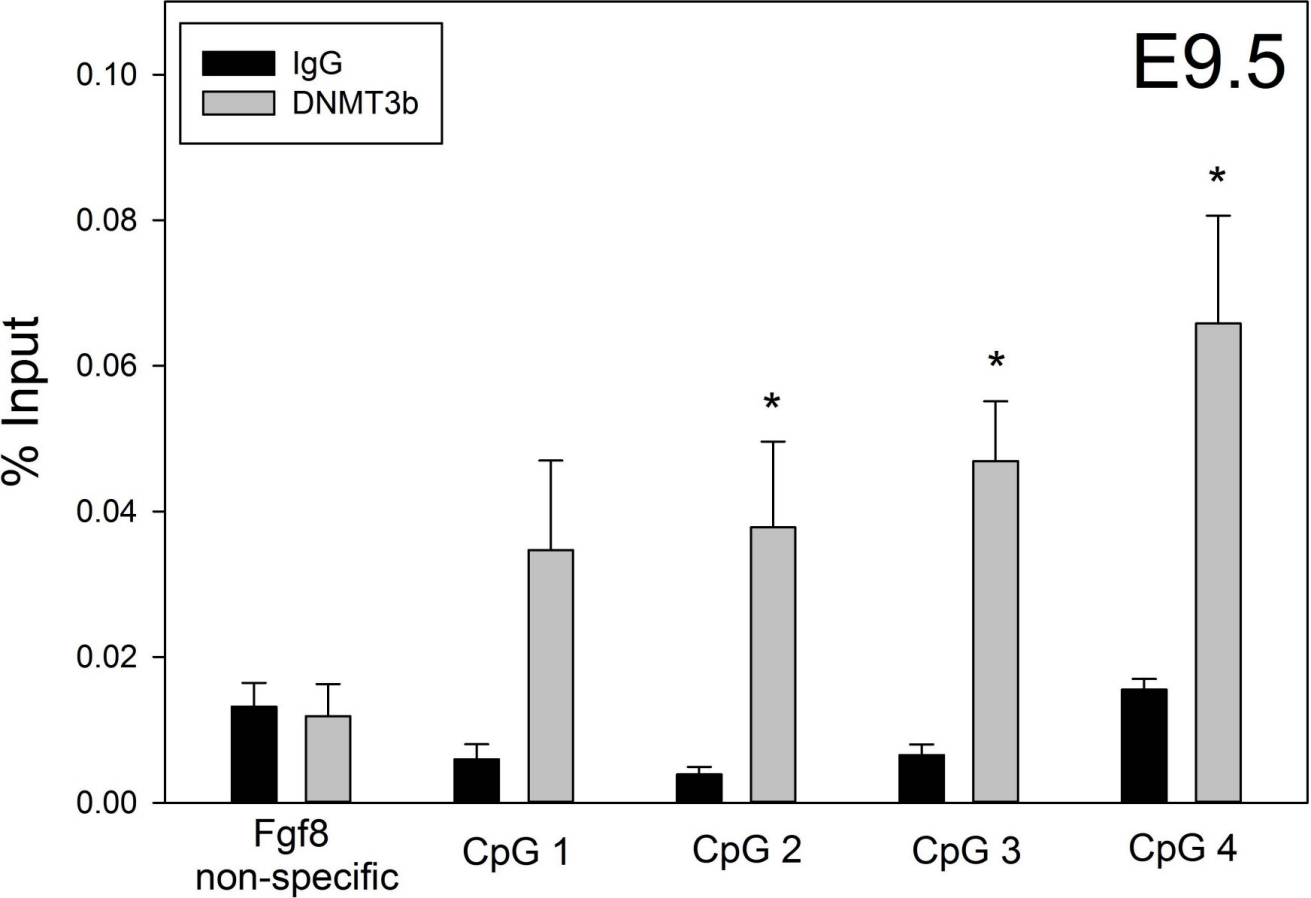
DNMT3b binds to the *Fgf8* promoter early in OP development. DNMT3b ChIP-RT-qPCR of 6 pooled E9.5 mouse OPs on the *Fgf8* promoter (n = 4). * indicates *p* < 0.05. Student’s *t*-test.

Our AZA treatment findings suggested that *Fgf8* expression was under the control of repressive DNA methylation in both the GT1-7 cell line and E10.5 OP. To explore this further, we tested the ability of DNMT proteins to associate with the *Fgf8* locus. While DNMT3a and DNMT1 were not bound to the *Fg8* locus at E9.5 or E13.5, DNMT3b was significantly enriched at E9.5 on CpG 2 (*p* = 0.03), CpG 3 (*p* = 0.03) and CpG 4 (*p* = 0.03), which are closest to the *Fgf8* TSS. In contrast, DNMT3b was not significantly enriched on CpG 1 (*p* = 0.06) and a non-specific region (*p* = 0.8) (Fig 4).

### 5hmC accumulation on the *Fgf8* promoter is driven in a time-dependent fashion

**Fig 5:**
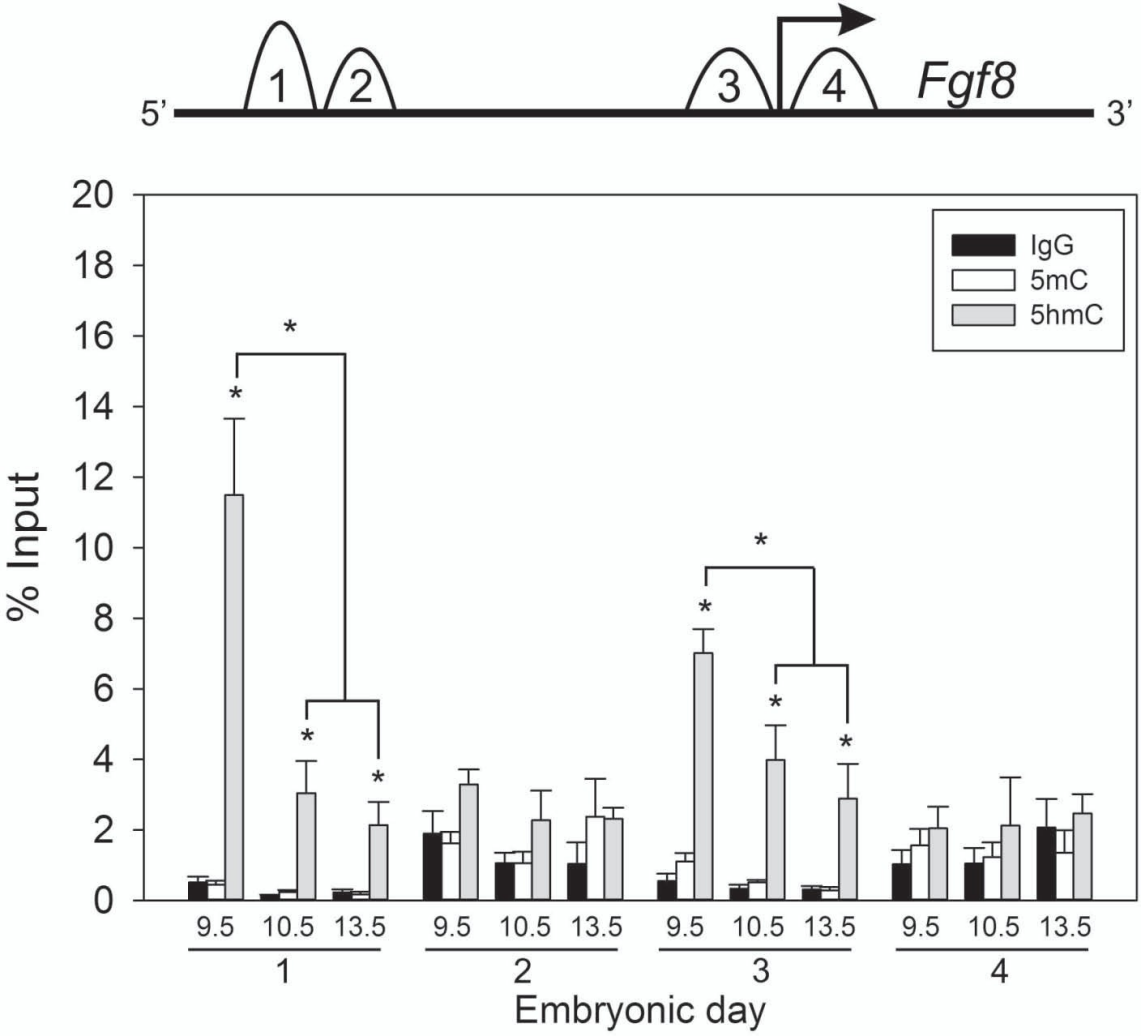
5hmC accumulation on the *Fgf8* promoter is driven in a time-dependent fashion. MeDIP RT-qPCR in 3–5 pooled mouse OPs at E9.5 (n = 4), E10.5 (n = 4), E13.5 (n = 4) along the *Fgf8* promoter. CpG islands are indicated in numbers 1–4. * indicates *p* < 0.05 compared to IgG; Student’s *t*-test. ** indicates *p* < 0.05 5hmC enrichment on CpG 1 and 3 between E9.5 and E10.5 or E13.5; One-way ANOVA followed by Holm-Sidak *post hoc*.

Here, we analyzed 5mC and 5hmC occupation along the *Fgf8* promoter region in developing OPs. Because our previous results indicated rapid changes in the expression levels of epigenetic modifiers (i.e., increased *Tet* and *Dnmt3a* and reduced *Dnmt3b* mRNA levels), we hypothesized that these enzymes may play a prominent role in regulating *Fgf8* transcription. We performed MeDIP using OPs from E9.5, E10.5 and E13.5 embryos. We found that 5hmC was enriched at 2 of the 3 CpG islands found along the promoter region of *Fgf8* (Fig 5). Individual Student’s *t*-tests comparing 5hmC levels within CpG 1 or CpG 3 found that 5hmC was highly enrichment at all ages (E9.5, E10.5, E13.5) when compared to IgG (*p* = 0.002, *p* = 0.03, *p* = 0.03; *p* = 0.0001, *p* = 0.03, *p* = 0.04; Fig 5). These results are in line with E8.5 frontonasal prominence tissue (i.e., pre-placodal) MeDIP data detecting significant 5mC and 5hmC enrichement on CpG 1 (*p* = 0.01; *p* = 0.002), and CpG 3(*p* = 0.0006; *p* = 0.0006) (S3 Fig). Interestingly, this observation coincided with the embryonic time period where OP *Fgf8* mRNA expression is high. One-way ANOVAs showed a significant decrease in 5hmC with time within CpG 1 (*p* = 0.002) and CpG 3 (*p* = 0.0001). In contrast, no significant enrichment of 5hmC was detected on CpG 2 or CpG 4 (*p* = 0.1, *p* = 0.2, *p* = 0.1; *p* = 0.2, *p* = 0.5, *p* = 0.7), and 5mC was not enriched at any of the 4 CpG sites (*p* = 0.7, *p* = 0.1, *p* = 0.6; *p* = 0.7, *p* = 1, *p* = 0.3; *p* = 0.2, *p* = 0.2, *p* = 0.9; *p* = 0.4, *p* = 0.8, *p* = 0.5, listed as age groups within primer sets, CpG 1–4, respectively.) (Fig 5). These results were verified using 2 different antibodies against 5mC and 5hmC.

### TET1 interacts with CpG 1 and CpG 3

**Fig 6:**
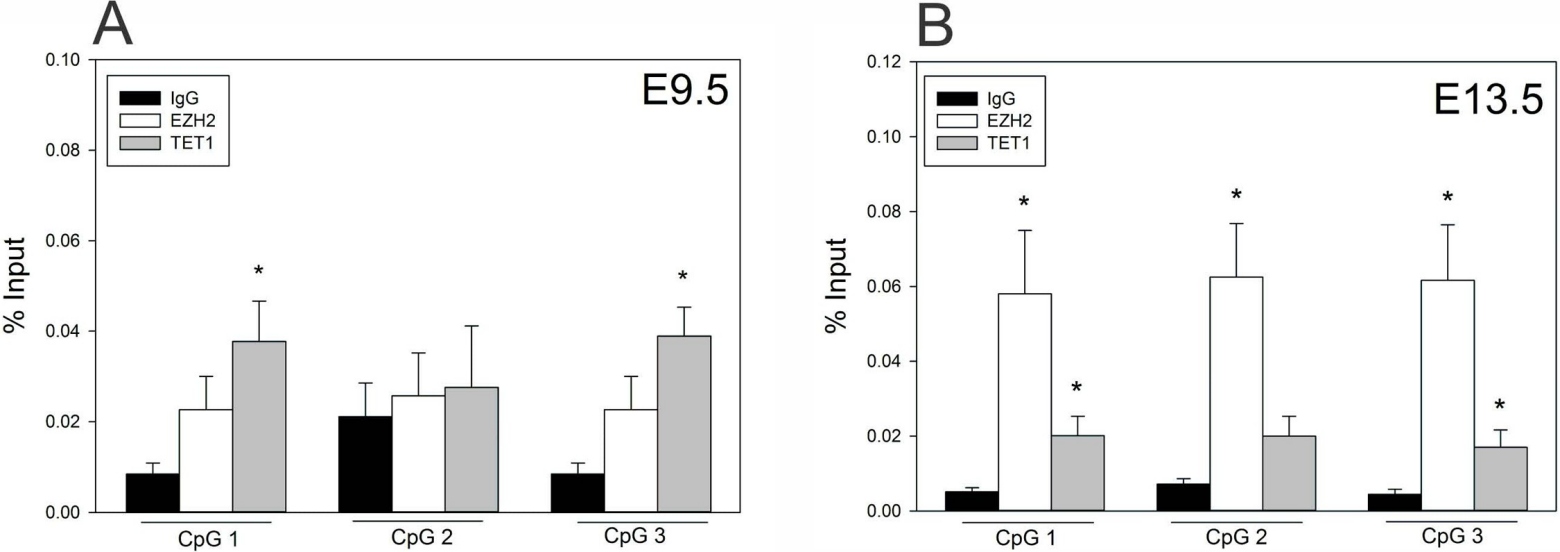
Epigenetic switch on the *Fgf8* promoter in the E9.5 and E13.5 OP. A) At E9.5 (n = 4), TET1 was enriched at CpG 1 and 3. B) At E13.5 (n = 4), TET1 was enriched at CpG 1 and 3, while EZH2 was enriched at all 3 CpG sites. * indicates *p* < 0.05; Student’s *t*-test.

Because we found significant enrichment of 5hmC on CpG 1 and CpG 2, we hypothesized that TET proteins are responsible for the conversion of 5mC to 5hmC on the *Fgf8* promoter. Specifically, we focused on TET1, since it is associated with gene promoters while TET2 is associated with gene bodies and near actively expressed exons [42]. Moreover, TET1 seems to play a role in the neuroendocrine system, as recent studies found that TET1-KO mice have impaired fertility, and TET1 expression responds to lutenizing hormone in gonadotropin cell lines [47,48]. Therefore, we performed ChIP to determine if TET1 co-localizes with 5hmC rich regions on the *Fgf8* promoter. Our results showed that at E9.5, TET1 localized to CpG 1 and 3, previously identified to be 5hmC enriched (*p* = 0.01, *p* = 0.004), and was absent on CpG 2 (*p* = 0.7) (Fig 6A). At E13.5, TET1 was signifigantly enriched only on CpG 1 (*p* = 0.03) and 3 (*p* = 0.04) (Fig 6B).

### Histone modifications regulate timing of *Fgf8* transcription

**Fig 7:**
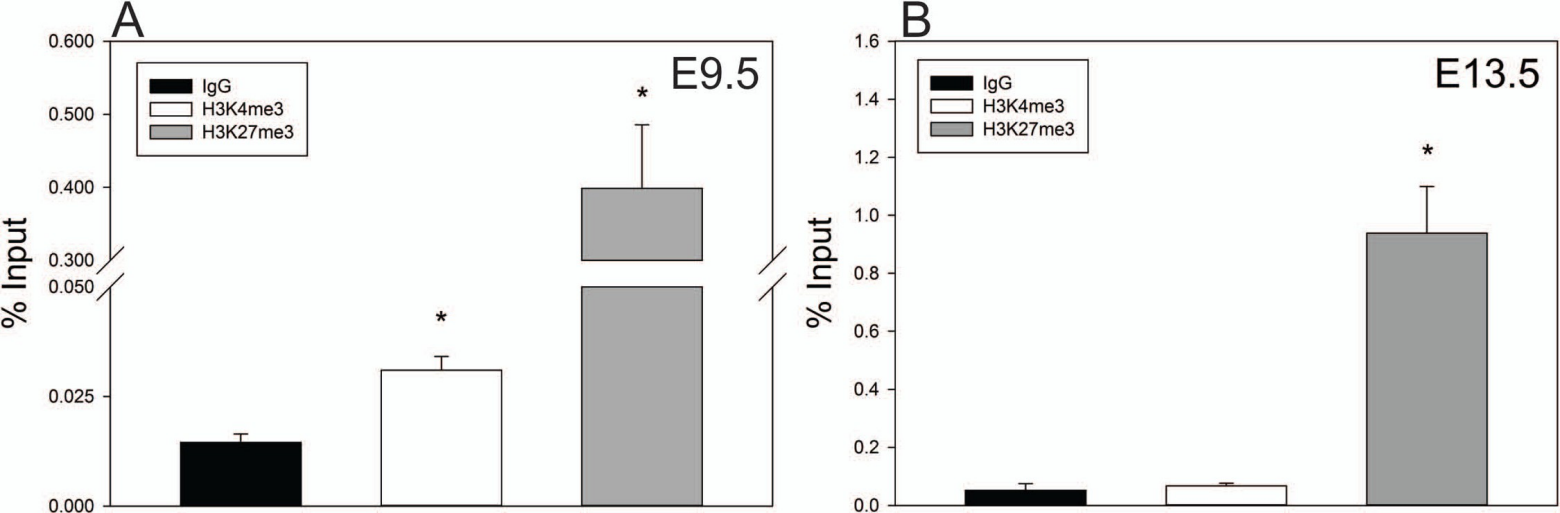
*Fgf8* histone modifications during GnRH neuron ontogenesis. A) ChIP for H3K4me3/H3K27me3 of 3–5 pooled E9.5 (n = 4) and B) 3–5 pooled E13.5 OP (n = 4) on CpG 3. E9.5 OPs are enriched for H3K4me3 and H3K27me3, whereas only H3K27me3 was detected in E13.5. * indicates *p* < 0.05; Student’s *t*-test.

Because TETs have been shown to functionally associate with PRC2 members and bivalent promoters, of which *Fgf8* is known to be part of [49–51], we hypothesized that PRC2-dependent histone modifications also contribute to *Fgf8* mRNA transcription. Indeed, our results demonstrate that at E9.5, *Fgf8* harbors both H3K4me3 and H3K27me3 near the TSS (*p* = 0.004, *p* = 0.004; Fig 7A), while at E13.5 only H3K27me3 is present (*p* = 0.002). H3K4me3 was not detected at E13.5 (*p* = 0.6); Fig 7B). We also tested whether PRC2 protein, EZH2, was recruited to the *Fgf8* promoter, and found that EZH2, which is responsible of H3K27 trimethylation, was enriched at CpG 1, 2, and 3 on the *Fgf8* promoter (*p* = 0.02, *p* = 0.008, *p* = 0.009; Fig 6B), which was not the case for E9.5 OPs (*p* = 0.1, *p* = 0.7, *p =* 0.1; Fig 6A).

### TET1 regulates *Fgf8* and *Fgfr1* transcription

**Fig 8:**
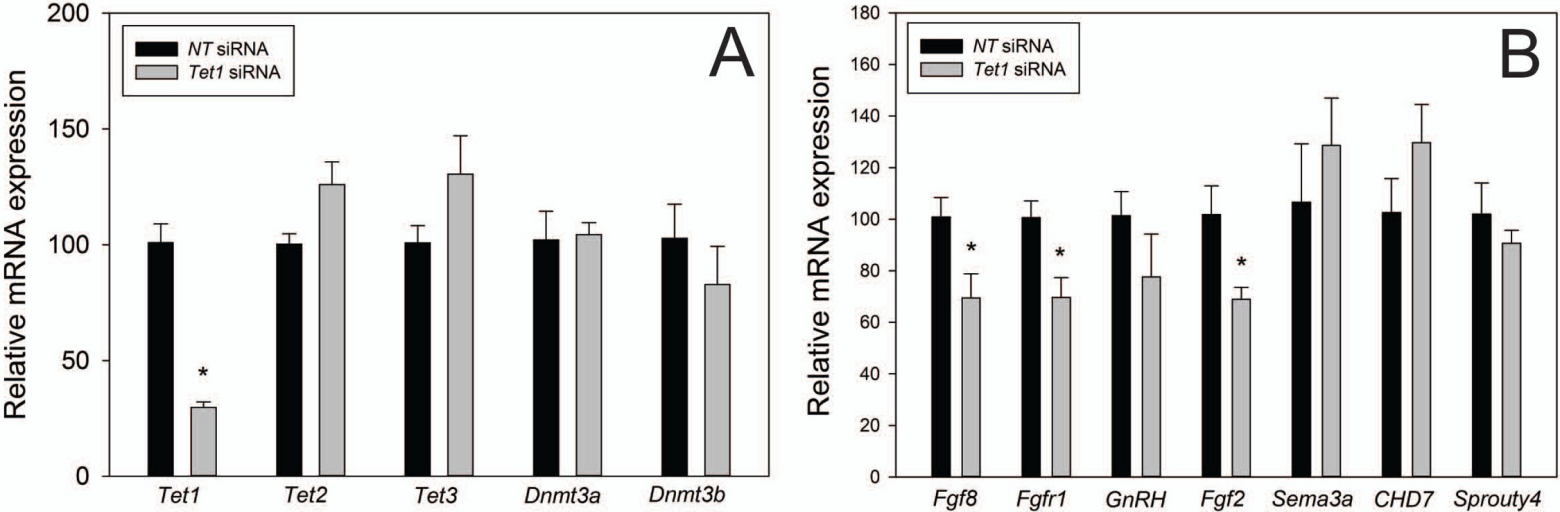
*Tet1* siRNA knockdown in GT1-7 neurons. A) *Tet1* siRNA (n = 4) did not affect other TET or DNMT mRNA expression, and reduced *Tet1* mRNA expression compared to *non-targeting* controls (n = 4). B) *Tet1* knockdown reduced *Fgf8*, *Fgfr1*, and *Fgf2* mRNA (n = 4). * indicates *p* < 0.05; Student’s *t*-test.

To determine the regulatory potential of TET1 on *Fgf8* mRNA expression, we used *Tet1* siRNA to reduce *Tet1* expression in GT1-7 neurons, which we previously showed to have higher levels of *Tet1* than GN11 neurons (Fig 3C). Accell *Tet1* siRNA experiments demonstrated a ~80% reduction in *Tet1* mRNA (*p* = 0.0002; Fig 8A) transcript compared to non-targeting control siRNA, and did not affect *Tet2* or *Tet3* mRNA (*p* = 0.05, *p* = 0.2). Accell *Tet1* siRNA-treated GT1-7 neurons showed a significant reduction in *Fgf8* and *Fgfr1* mRNA (*p* = 0.04, *p* = 0.02; Fig 8B). While GnRH mRNA trended down, it was not signifigant (*p* = 0.1).

## Discussion

Timing of gene expression during development is critical for proper onset of neuronal systems and embryonic patterning. In the present study, we show that *Fgf8* transcription is temporally regulated via TET1 during development of the GnRH system in the embryonic mouse OP. We also found that TET1 continued to interact with specific CpG-rich regions on the *Fgf8* promoter after emergence of GnRH neurons. Interestingly, we found that these TET1-interacting *Fgf8* promoter regions also recruited EZH2, which likely lead to the observed increased H3K27 trimethylation. Taken together, these sequential epigenetic events suggest that TET1 maintains the *Fgf8* promoter in a hypomethylated state, while subsequent recruitment of members of the PRC2 complex promote the repressive actions of H3K27me3 on *Fgf8* transcription. These results underscore the importance of epigenetic-dependent timing of *Fgf8* expression during GnRH neuron emergence, and that disruptions in the epigenome not only disrupt *Fgf8* signaling but may also play a critical role that results in KS pathogenesis.

CpG dinucleotide methylation is largely determined by the activity of DNMTs. While DNMT1 was not significantly upregulated during OP development, DNMT3a was upregulated ten-fold, while DNMT3b was downregulated in the E13.5 OPs as compared to E10.5 OPs. Additionally, we found enrichment of 5mC on the *Fgf8* promoter at CpG 1 and CpG 3 in E8.5 frontonasal promininces (S3 Fig), indicating a role for DNMTs. Previous studies have shown DNMT3a is detected primarily in post-mitotic olfactory receptor neurons, while DNMT3b expression was restricted to proliferating progenitor neurons, underlining the possibility that GnRH progenitors or their neural progenitors may, in part, rely on epigenetic machinery as a mechanism for neuronal differentiation [52]. In support, we found that DNMT3b was bound near the TSS and at an intragenic site of the *Fgf8* promoter at E10.5, but devoid of enrichment at E13.5. When E10.5 OP explants were treated with AZA, *Fgf8* transcription increased, indicating at E10.5, *Fgf8* transcription is under the repressive effects of DNMTs, presumably by DNMT3b at the gene body. This assumption is based on our observations that only DNMT3b was found to interact with CpG 3 and CpG 4, which are proximally located near the *Fgf8’*s TSS and 5’ exon/gene body. Moreover, localization of DNMT3b at the intragenic CpG 4 region on the actively transcribed *Fgf8* locus may further suggest CpG 4 is involved with mRNA processing, such as elongation, splicing, or recruitment of other DNA binding proteins [53–56].

Timing of gene expression during embryonic brain development is imperative during fate specification of neuronal populations, of which *Fgf8* is critically important. During development of the OP, we found higher expression in *Tet1-3* mRNA at E13.5. Furthermore, dot blot analysis showed higher levels of genome-wide hydroxymethylation at E13.5. In the OP, we found a clear relationship between the abundance of *Fgf8* mRNA transcripts and 5hmC levels on CpG 1 and 3 of the *Fgf8* promoter. These two 5hmC-rich regions were also co-occupied by TET1, demonstrating that TET1 is likely responsible for converting 5mC to 5hmC. These results indicate that TET1 discretely controls the timing of *Fgf8* expression during development of GnRH neurons to ensure proper FGF8 signaling. Interestingly, we also found high 5hmC levels on the *Fgf8* promoter in midbrain-hindbrain tissue at E10.5 (S4 Fig), which may suggest that TET-dependent demethylation is a general mechanism for *Fgf8* transcription in neuronal populations.

TET-catalyzed demethylation also affects nearby nucleosome compaction by modifying histone methylation status [57–60]. Aside from catalyzing the conversion from 5mC to 5hmC, TET1 can also maintain histone bivalency [50,61–63], which we hypothesized contributes to timing of *Fgf8* expression. We therefore measured H3K4 and H3K27 trimethylation levels in the developing OP. We found that at E9.5, the *Fgf8* TSS is associated with H3K4me3 and H3K27me3. In contrast, at E13.5, the *Fgf8* TSS exclusively harbors H3K27me3, suggesting that H3K27me3 has a repressive role on *Fgf8* transcription. Based on previous studies, we infer that this histone switch in H3K4 and H3K27 trimethylation promotes nucleosome compaction, and is therefore responsible for the time-dependent downregulation of *Fgf8* transcription in the embryonic mouse OP [51,64,65].

Our ChIP studies support that TET1 interaction with the *Fgf8* promoter is rather stable and showed continuous interaction with the *Fgf8* promoter. We also found that EZH2, which is highly expressed in the E9.5 and also in the E13.5 OP (S5 Fig), was enriched on the E13.5, but not on the E9.5 *Fgf8* promoter, suggesting that TET1 may help recruit the PRC2 complex to trimethylate H3K27 at these *Fgf8* promoter regions. Indeed, previous studies have found that TET1 protein can directly interact with EZH2 [64,66]. Taken together these data suggest that TET1 is not only responsible for DNA demethylation, but may also be responsible for subsequent HK27-specific trimethylation through the recruitment of EZH2, as suggested in earlier studies in mouse embryonic stem cells [64,65]. It is also possible that TET1 is replaced by EZH2 during periods of high *Fgf8* transcription, which will repress *Fgf8* transcription by depositing H3K27me3, thereby contributing to the transient nature of *Fgf8* expression in the developing mouse OP. Alternatively, recent biochemical studies indicate a feedback mechanism by which *de novo* RNA transcripts (including *Fgf8* transcripts) contribute to EZH2 recruitment by interacting with the RNA binding domain of EZH2 [67]. In this mechanism, mRNA production is inhibited by recruiting other PRC2 core proteins to gene promoters [68–70]. Currently, more studies are needed to pinpoint which of these mechanisms is the most likely pathway that contributes to transcription of *Fgf8* in the embryonic mouse OP.

Previous studies in GT1-7 neurons indicated that direct TET function, specifically TET2, is required for *GnRH* mRNA transcription and affected histone methylation status on the *GnRH* promoter [71]. Here, our *Tet1* siRNA experiments demonstrated that TET1 is required for regulating *Fgf8* transcription, a molecular event that we previously showed to be required for the emergence of the GnRH neuronal system in mice [3,72]. Therefore, we conclude that TET1 binding to the *Fgf8* promoter in OP progenitor cells is not only critical for inducing *Fgf8* transcription but may also be required for the emergence of GnRH neurons. Additionally, genome-wide hydroxymethylation of DNA and RNA, mediated through TET enzyme activity is perhaps of equal importance during development of GnRH neurons. In support, our studies also showed that TET1 controls *Fgf2* and *Fgfr1* transcription in GT1-7 neurons. This possibility is not without merit given that earlier studies early showed GnRH neuronal development is a multi-genic process. Overall, our studies provide further evidence that upstream epigenomic regulators are involved in GnRH neuron differentiation and the onset of KS.

**Fig 9:**
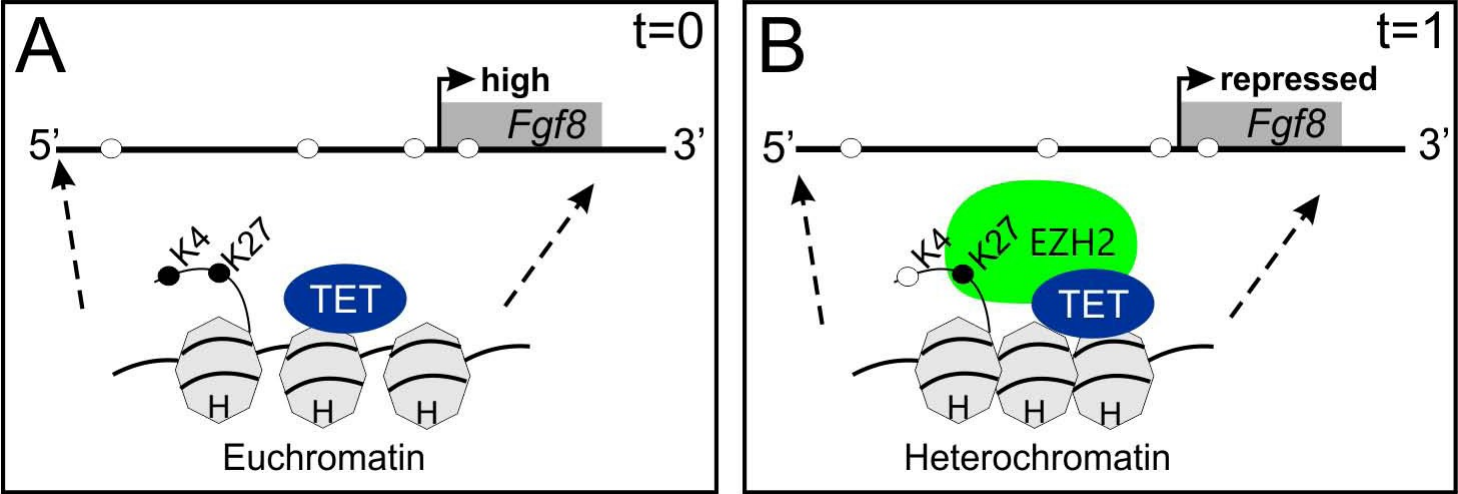
Schematic model of age-dependent *Fgf8* transcriptional control in the embryonic mouse OP. A) At time (t) = 0, TET1 interaction with the *Fgf8* promoter maintains its hypomethylated, and H3K4me3/H3K27me3 bivalent state, thereby inducing *Fgf8* transcription B) In contrast at t = 1, EZH2 recruitment maintained H3K27 trimethylation, while H3K4me3 was lost, which represses *Fgf8* transcription. Closed circles = methylated, open circles = demethylated.

In conclusion, the present study provides evidence that *Fgf8* is transcriptionally regulated by TET1. We show that TET1 maintains a *Fgf8’s* hypomethylated state, which decreases with embryonic age. Moreover, TET1 together with EZH2 likely maintains a dynamic bivalent histone state of the chromatin proximal to the *Fgf8* locus that contributes to the transient nature of embryonic *Fgf8* expression. These and other studies largely point to *Fgf8* activation through dynamic chromatin arrangement and specific methylation events (Fig 9). Furthermore, DNA demethylation and the conversion of 5mC to 5hmC, play an integral role in *Fgf8* transcription during development of the GnRH system. Overall, disruptions in the pre-hypothalamic epigenome could have major consequences on the *Fgf8* signaling system and GnRH neurodevelopment, resulting in congenital hypogonadotropic hypogonadism disorders, such as KS.

## Supporting information

S1 Fig Negative MeDIP qPCR in 3–5 pooled mouse OPs at E9.5 on the 3’UTR *Fgf8*. Negative control region in comparison to E9.5 CpG1 site (n = 4). * indicates *p* < 0.05; Student’s *t*-test.
(TIF)

S2 Fig Propidium iodine staining in GT1-7 cells treated with AZA for 3 days *in vitro* (n = 3); One-way ANOVA. (TIF)

S3 Fig Significant levels of 5mC on the *Fgf8* promoter at E8.5. MeDIP qPCR in 8–10 pooled mouse frontonasal prominences at E8.5 along the promoter of *Fgf8* CpG 1 and CpG 3 (n = 4). * indicates *p* < 0.05; Student’s *t*-test.
(TIF)

S4 Fig *Fgf8* promoter landscape in the E10.5 Midbrain-Hindbrain. MeDIP qPCR in 3–5 pooled mouse midbrain-hindbrains at E10.5 along the promoter of *Fgf8* CpG islands indicated in numbers 1–3 (n = 4). * indicates *p* < 0.05; Student’s *t*-test.
(TIF)

S5 Fig EZH2 is highly expressed in the developing OP. A). *EZH2* mRNA expression in the E9.5 versus E13.5 OP; * indicates *p* < 0.05; Student’s *t*-test.
(TIF)
