# Supplementary figures and images for: TET1 regulates fibroblast growth factor 8 transcription in gonadotropin releasing hormone neurons
Source: PLoS One. 2019 Jul 30;14(7):e0220530. doi: 10.1371/journal.pone.0220530 (PMC6667164; doi:10.1371/journal.pone.0220530)

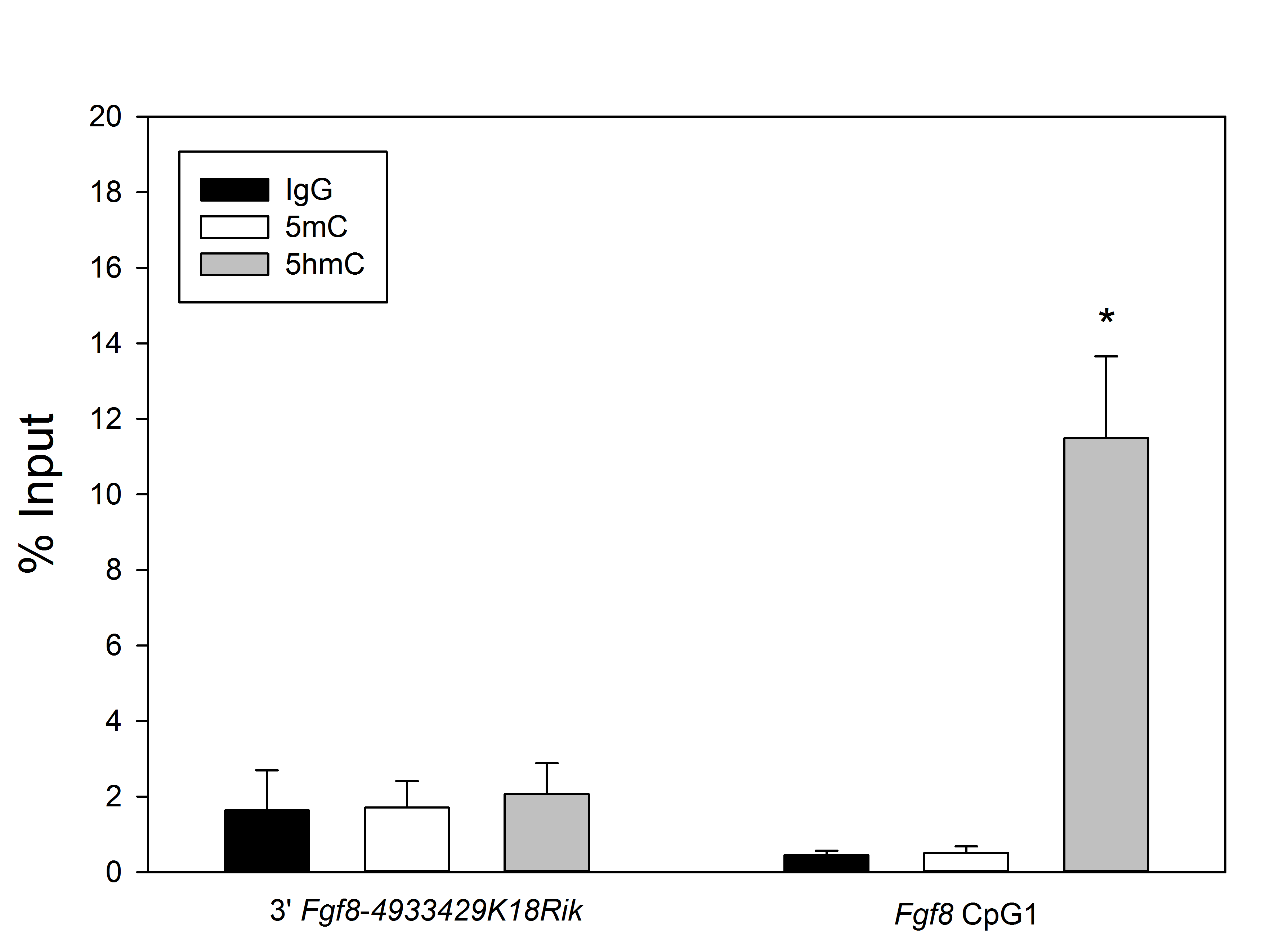

Supplement: S1 Fig — Negative control region in comparison to E9.5 CpG1 site (n = 4). * indicates p < 0.05; Student’s t-test. (TIF) [file pone.0220530.s001.TIF]

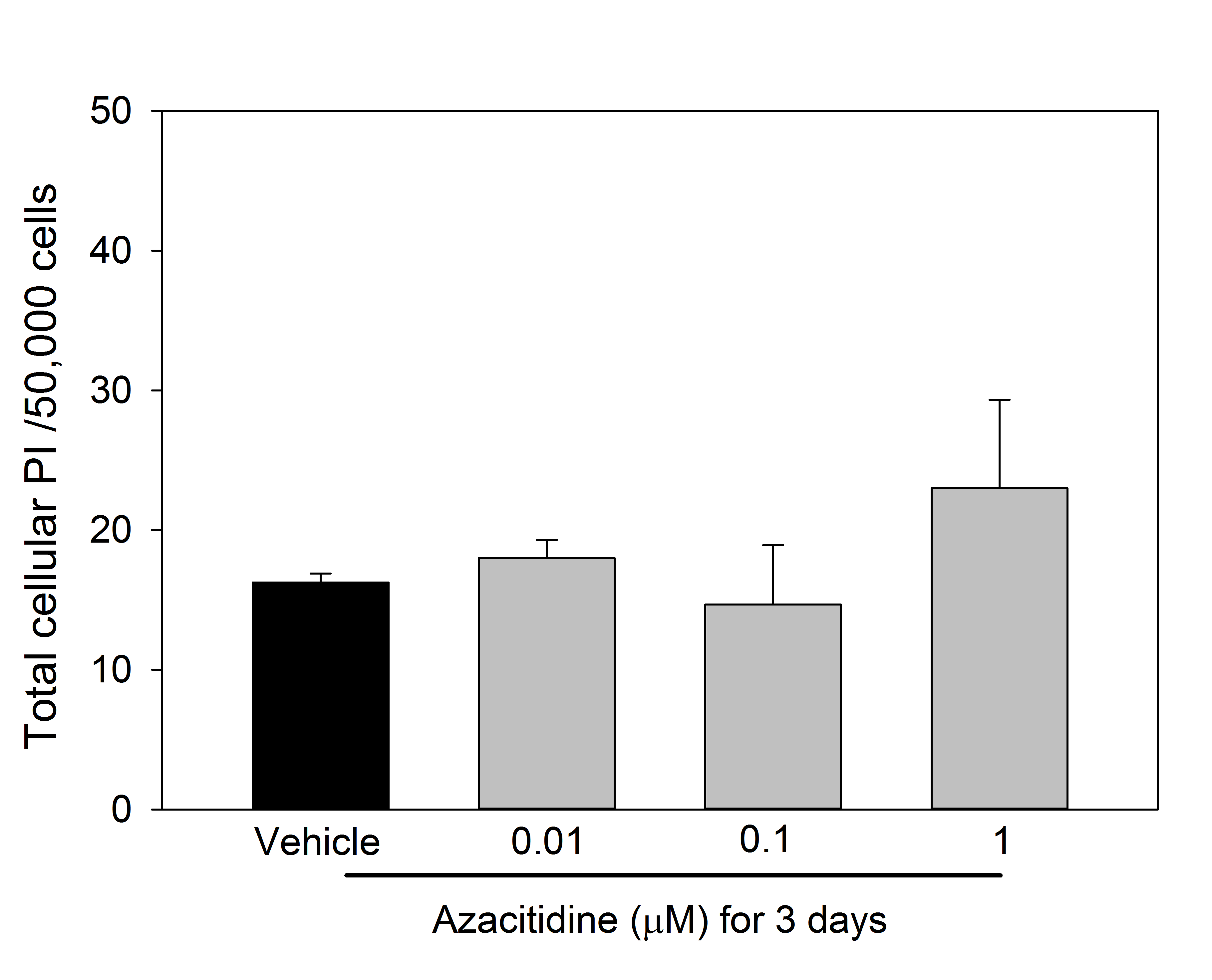

Supplement: S2 Fig — (TIF) [file pone.0220530.s002.TIF]

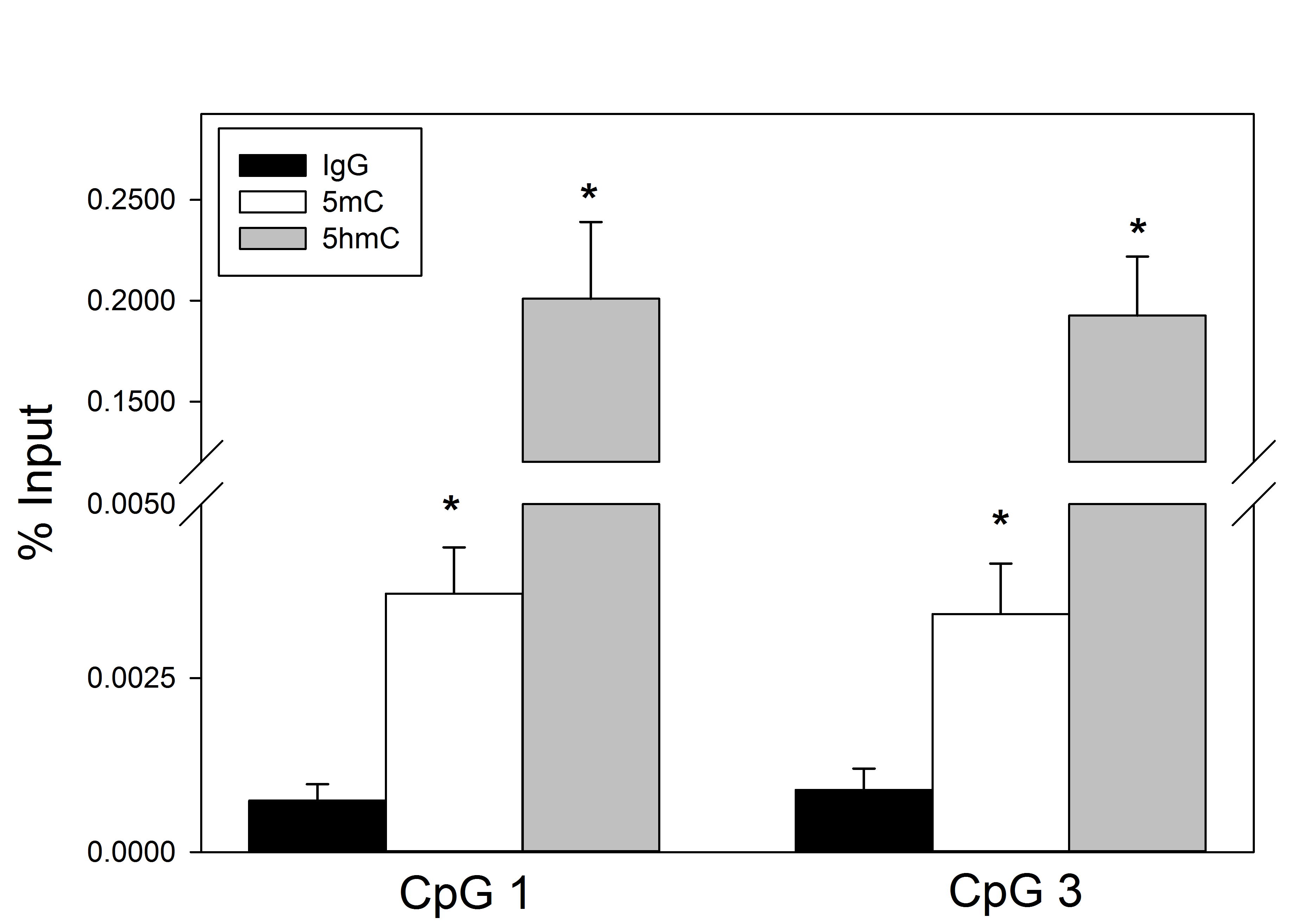

Supplement: S3 Fig — MeDIP qPCR in 8–10 pooled mouse frontonasal prominences at E8.5 along the promoter of Fgf8 CpG 1 and CpG 3 (n = 4). * indicates p < 0.05; Student’s t-test. (TIF) [file pone.0220530.s003.TIF]

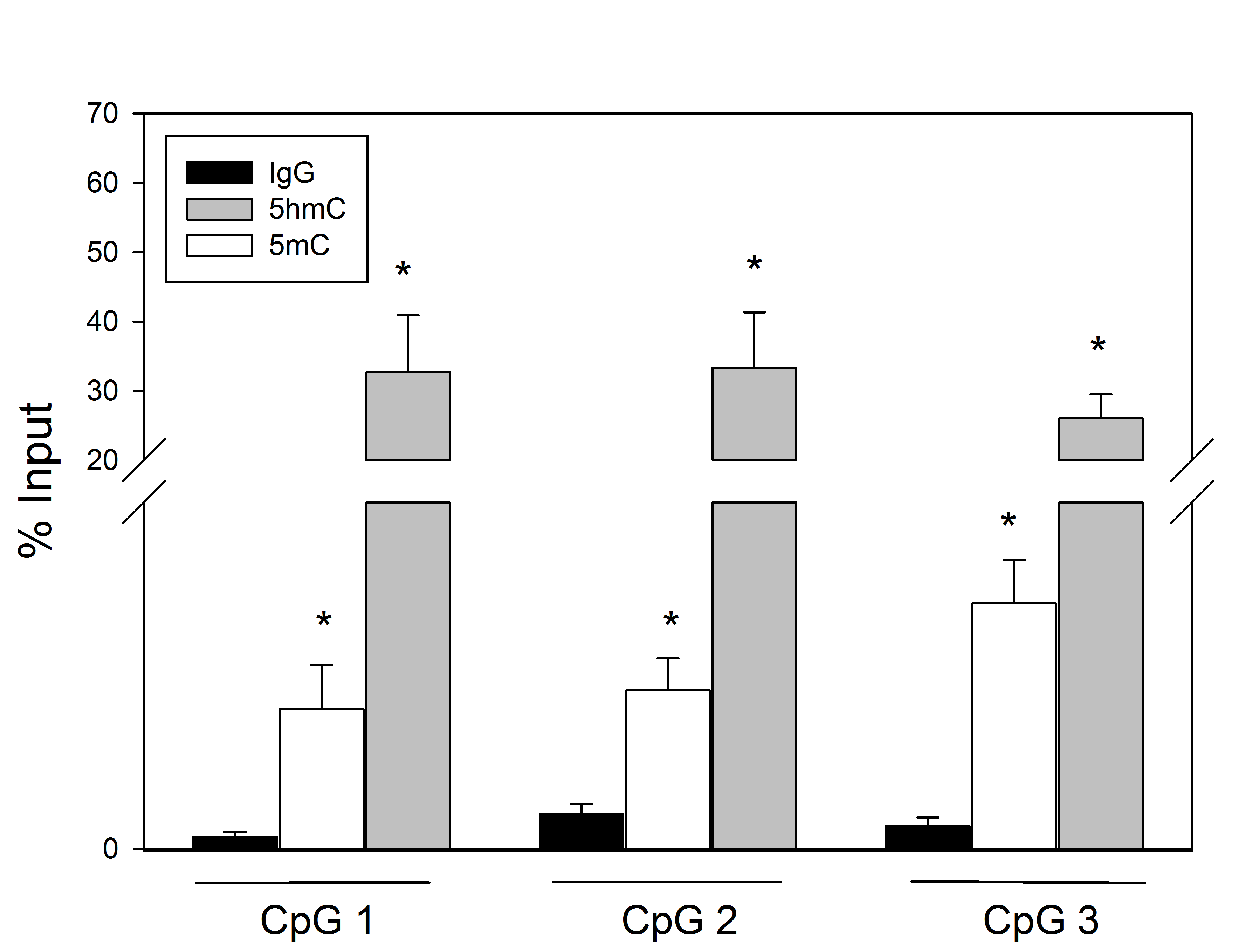

Supplement: S4 Fig — MeDIP qPCR in 3–5 pooled mouse midbrain-hindbrains at E10.5 along the promoter of Fgf8 CpG islands indicated in numbers 1–3 (n = 4). * indicates p < 0.05; Student’s t-test. (TIF) [file pone.0220530.s004.TIF]

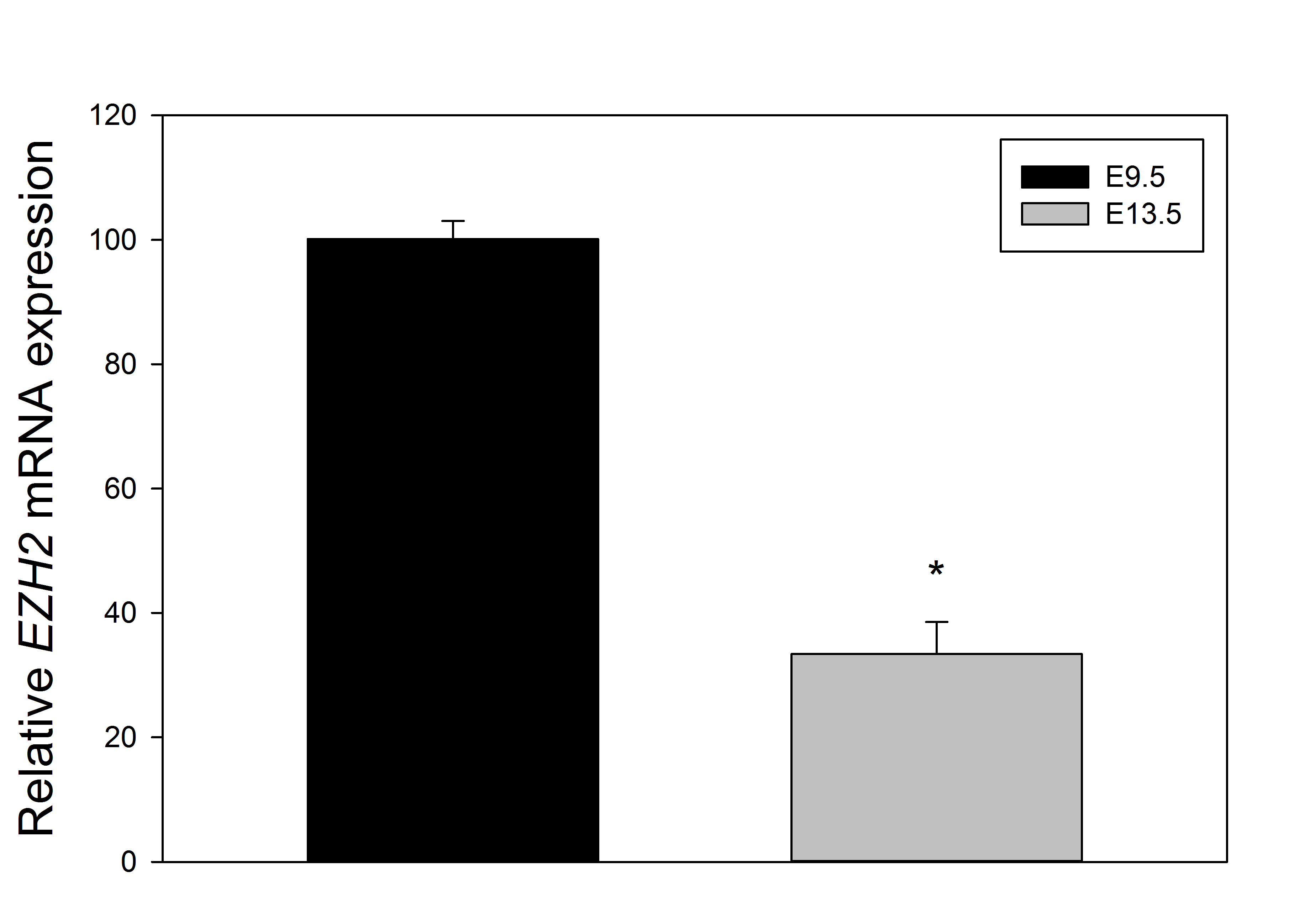

Supplement: S5 Fig — A). EZH2 mRNA expression in the E9.5 versus E13.5 OP; * indicates p < 0.05; Student’s t-test. (TIF) [file pone.0220530.s005.TIF]
